# Validation of a German version of the Caregiver Quality of Life Index-Cancer (CQOLC) in a sample of significant others of breast and gynaecologic cancer patients

**DOI:** 10.1186/s41155-020-00155-8

**Published:** 2020-07-20

**Authors:** Anna Tamara Ehmann, Cornelia Mahler, Nadja Klafke

**Affiliations:** 1 Institute of Occupational and Social Medicine and Health Services Research, University Hospital Tübingen, 72074 Tübingen, Wilhelmstraße 27 Germany; 2 Department of Nursing Science, University Hospital Tübingen, Hoppe-Seyler-Straße 9, 72076 Tübingen, Germany; 3 Department of General Practice and Health Services Research, University Hospital Heidelberg, Im Neuenheimer Feld 130.3, 69120 Heidelberg, Germany

**Keywords:** Cancer, Caregiver, Support, Quality of life, Questionnaire, Validation, Reliability, Cancer care

## Abstract

There is no disease-specific instrument to measure the quality of life of significant others of cancer patients in Germany. In this study, we evaluated the reliability and construct validity of a German version of the Caregiver Quality of Life Index-Cancer (CQOLC) in a sample of 212 caregivers of breast and gynaecologic cancer patients. The CQOLC was administered along with the World Health Organization Quality of Life short version (WHOQOL-BREF) to caregivers of patients taking part in a randomized-controlled intervention study. Data of 212 caregivers were gained at the baseline of the study. Internal consistency was determined by Cronbach’s α. Construct validity was examined by conducting a confirmatory factor analysis (CFA) and hypothesis testing. Correlations between change scores with patients’ global health-related quality of life (HRQoL) were calculated for three time points to evaluate the responsiveness. The three subscales “burden”, “disruptiveness”, and “financial concerns” indicate to a good reliability of the instrument (Cronbach’s α ranged between 0.754 and 0.832), while the subscale “positive adaptation” demonstrated low reliability (*α* = 0.579). A CFA based on data from the whole set of CQOLC items resulted in CFI levels < .90, and a CFA without problematic items resulted in CFI levels also < .90.

The construct validity of the CQOLC could be approved by a moderate to high convergence with close variables as the global HRQoL. Mean differences between caregivers of curatively or palliatively treated patients were nonsignificant (*p* = 0.959) at T1. Correlations for responsiveness were low with correlation coefficients ranging from 0.030 to 0.326. These data indicate that additional research is needed to further verify the validity of the instrument. The German scale of the CQOLC might be appropriate for clinical and research use, if the wording of some items is refined and if content validity is also assessed by caregivers themselves. The assessment of cancer patients’ caregiver’s quality of life can contribute to a better understanding of the effects of patient-oriented interventions including also closely involved next of kin’s around the cancer patients.

## Introduction

In Germany, the incidence of cancer has almost doubled since the 1970s. In addition, the number of patients surviving the disease has increased even more up to four million cases with subsequent implications for patients requiring follow-up care within the German healthcare system (Barnes et al., 2016). Patients who receive cancer treatment have to deal with various psychological and physiological symptomatic burdens (Wagland et al., 2015). Negative effects resulting from the diagnosis and treatment of cancer are not only experienced by patients, but also by their family members closely involved in patients’ daily activities interrupted by the cancer diagnosis and its treatment (Lewis, 1990; Nijboer, Tempelaar, Sanderman, Triemstra, & Spruijt, 1998). By providing frequent support, close persons around the cancer patients also experience burden such as increased psychological distress, physical symptoms, and changes in daily routines (Lewis, 1990) leading to a decline in their quality of life (Nijboer et al., 1998). The quality of life experience of patients and their family members indicates to dyadic effects (Weitzner, Jacobsen, Wagner, Friedland, & Cox, 1999). Therefore, it is crucial that also the needs of cancer patients’ family members are addressed within supportive and follow-up healthcare programmes, and that their disease-specific quality of life is measured accordingly; therefore, a specific assessment is highly needed. According to the World Health Organization (1997), HRQoL is a broad and complex concept influenced by individual physical health, psychological condition, social relationships, and personal beliefs as well as environmental factors.

For the assessment of the overall impact of cancer on the HRQoL perceived by the caregiver, Weitzner et al. (1997) developed the Caregiver Quality of Life Index-Cancer (CQOLC). This instrument consists of 35 items and four factors. The assessment instrument has been validated in the original American English version (M. A. Weitzner & McMillan, 1999) as well as in other languages. In addition to validation studies conducted in Asian countries (Bektas & Ozer, 2009; Duan et al., 2015; Khanjari, Oskouie, & Langius-Eklof, 2012; Mahendran et al., 2015; Ozer, Firat, & Bektas, 2009; Rhee et al., 2005; Tang, Tang, & Kao, 2009), the CQOLC was used in Portugal (Santos, Ribeiro, & Lopes, 2003), Italy (Pugliese et al., 2004), and France (Lafaye, de Chalvron, Houede, Eghbali, & Cousson-Gelie, 2013). Validation studies were carried out resulted in variations in number of items and factor structure. A translated version in German of this instrument was provided by Mapi Research Trust (2017) in the Patient-Reported Outcome and Quality of Life Instruments Database (PROQOLID). However, no psychometric testing of the instrument had been performed and reported to date. To our knowledge, there was no other validated disease-specific instrument in German available to evaluate caregivers’ HRQoL.

### The present research

Our interest here was to evaluate the reliability and construct validity of the German version of the CQOLC. This validation study is based on data from caregivers of patients participating in the CONGO (*Co*mplementary Nursing in *G*ynecologic *O*ncology) study investigating the potential of complementary therapies in supportive care (Klafke et al., 2015).

The CONGO study included patients with breast and gynaecologic cancer who received a new regimen of chemotherapy recruited from two hospitals in South Germany. Patients were randomized into an intervention group and a control group; patients of the intervention group received complementary nursing interventions additionally to routine care. The primary outcome of the CONGO study was patients’ HRQoL assessed by means of the European Organization for Research and Treatment of Cancer Quality of Life Questionnaire (EORTC-QLQ-C30) (Aaronson et al., 1993), and the results of the primary patient-oriented outcome have been reported elsewhere (Klafke et al., 2019).

## Method

### Participants

Between July 31 2014 and February 9 2016, adult breast and gynaecologic cancer patients starting a new regimen of chemotherapy were invited to take part in the intervention study. After providing informed consent for participation, the participants (*N =* 297) were asked for their consent to get in touch with their main caregiver, i.e., a significant other person who provides most assistance in daily activities and patient care. Seventy-five patients (25%) did not give consent to contact a caregiver. The main reason for not giving permission to contact their caregivers was in order to protect them from additional burden. In total, 212 caregivers gave consent for participation in this study. This intervention study has been registered with German Clinical Trials Register under DRKS00006056 on 14 April 2014. Ethical approval was granted by the ethics committees of the University of Heidelberg (S-008/2014) and the State Medical Council of Baden-Wuerttemberg (B-F-2015-037), Germany. In addition, the study was performed in compliance with the principles outlined in the Declaration of Helsinki on ethical principles for medical research involving human subjects.

### Procedure

According to the protocol of the intervention study (Klafke et al., 2015), caregivers were asked to participate in the survey at three time points: First, at the baseline (T1), second, at the end of the intervention (T3, max. after 24 weeks), and third, in a follow-up assessment 6 months after the end of the intervention (T4). The CQOLC and the World Health Organization Quality of Life short version (WHOQOL-BREF) (Skevington, Lotfy, & O’Connell, 2004) were sent to the caregivers at T1, T3, and T4. A sociodemographic questionnaire complemented the survey at T1. All data of patients’ caregivers were collected parallel to the data collection of the patients receiving chemotherapy treatment. The caregivers could complete their questionnaires at home and sent them back by prepaid mail. By returning the questionnaire, the caregivers gave their consent to participate in the study.

### Materials

#### Caregiver Quality of Life Index-Cancer (CQOLC)

The CQOLC was developed by Weitzner et al. (1997) as a disease-specific self-reported HRQoL instrument for family caregivers of cancer patients. The development of the scale included semi-structured interviews with patient-caregiver dyads and healthcare providers for item generation. The original version of the CQOLC consists of 35 items (see Additional file 1) which are measured on a5-point Likert-type scale (0 “not at all “to 4” very much”). A higher score indicates better HRQoL. In addition to a total score achieved by summing up 35 (range from 0–140 for the overall scale), there are four subscales: *burden* (10 items), *disruptiveness* (7 items), *positive adaptation* (7 items), and *financial concerns* (3 items). The other 8 items (item 2, 4, 13, 15, 23, 30, 32, 35) do not load on one of these factors according to the unpublished manual by the author. The composite measurement scale asks about the condition throughout the previous 7 days. Weitzner et al. (ebd.) report good results for the reliability of the scale. Test-retest reliability was 0.95, and internal consistency for the total scale was 0.90. The subscales had reliability values of *α* = 0.89 (*burden*), *α* = 0.83 (*disruptiveness*), *α* = 0.73 (*positive adaptation*), and *α* = 0.81 (*financial concerns*). We decided to apply this instrument, as another evaluation study of Weitzner, Jacobsen, et al. (1999) demonstrated that the CQOLC measures substantially different aspects than general HRQoL instruments, and values of convergent and divergent validity have been satisfactory as demonstrated with the Medical Outcomes Study Short Form 36 (MOS SF-36) and the Caregiver Burden Scale. We considered another advantage of applying the CQOLC, as this instrument is also able to differentiate between caregivers of curatively and palliatively treated cancer patients (Weitzner, McMillan, & Jacobsen, 1999). Other advantages of the CQOLC have been confirmed by a systematic review (Edwards & Ung, 2002).

In the German version of the CQOLC received from Mapi Research Trust in 2017 (see Additional file 1), which had undergone forward and backward translation, one item (item 4, question about satisfaction with sexual life) had been excluded. This resulted in the calculation of the total score on the basis of 34 items.

#### World Health Organization Quality of Life (WHOQOL-BREF)

While the CQOLC is a disease-specific HRQoL instrument, the WHOQOL-BREF (Skevington et al., 2004) is an instrument asking for global HRQoL in terms of the previous 4 weeks. It consists of 26 items. The first two items ask about general HRQoL. The other 24 items consist of 4 subscales. The domains are *physical health* (7 items), *psychological* (6 items), *social relationships* (3 items), and *environment* (8 items). In a cross-cultural study, the psychometric properties were verified (Skevington et al., 2004). The German version reached good values for internal consistency in all domains in a sample of *N =* 2408: physical domain *α* = 0.88, psychological domain *α* = 0.83, social domain *α* = 0.76, and environmental domain *α* = 0.78 (Skevington et al., 2004). In terms of the assessment of HRQoL, the WHOQOL-BREF is considered as a standard instrument internationally (Bullinger & Schmidt, 2006). The advantages of the WHOQOL-BREF consist in the cross-cultural sensitive concept and the availability in international major languages (Skevington et al., 2004) as well as in norm scores (Mokkink et al., 2010b). The calculations of the sub scores used in this study followed the official manual (Angermeyer, Kilian, & Matschinger, 2000).

#### European Organization for Research and Treatment of Cancer (EORTC-QLQ-C30)

The EORTC-QLQ-C30 (Aaronson et al., 1993) was used for the assessment of the primary outcome of the complex healthcare intervention tested in the randomized-controlled intervention study (Klafke et al., 2015; Klafke et al., 2016). This instrument is specific for oncological studies and asks for the conditions in the past week (Aaronson et al., 1993). As it is an instrument that is standardized, psychometrically tested and validated in different languages, the EORTC-QLQ-C30 has been established as a reliable and user-optimized measurement system (Bullinger & Schmidt, 2006). There are two items for the assessment of global HRQoL. With the other 28 items, five functional scales and nine symptom scales can be calculated. Six separate items request further information about physical symptoms and financial concerns. The global HRQoL items have a 7-point Likert-scale, whereas the other items have a 3-ranged Likert-scale.

### Statistical analysis

We followed the Consensus-based Standards for the selection of health status Measurement Instruments (COSMIN) checklist (Mokkink et al., 2010; Mokkink et al., 2010a; Mokkink, Terwee, Patrick, et al., 2010b). After consulting M. Weitzner, missing values were substituted by the mean. In our validation study, we used the analytical concepts of classical test theory. Confirmatory factor analysis (CFA) with maximum likelihood estimation was carried out with SPSS AMOS 24. All analyses were computed using the IBM SPSS software package (Version 24).

#### Reliability

Internal consistency was computed with Cronbach’s alpha. A minimum of 0.7 is considered to be satisfactory (Nunnally & Bernstein, 1994). The Spearman-Brown coefficient with a first (items 1–17) and a second part (items 18–34) of the instrument was calculated. A coefficient above 0.7 is considered acceptable (Crocker & Algina, 1986; Lord & Novick, 1968).

#### Construct validity

The match between the model of the original factor structure and the collected data can be assessed based on various fit statistics by performing a confirmatory factor analysis (CFA). The goodness of model fit was examined with the indices chi square value divided by degrees of freedom (CMIN/df), root mean square error of approximation (RMSEA), and Bentler Comparative Fit Index (CFI). A model fit is suggested to be acceptable if *χ*^2^/df is ≤ 2, RMSEA ≤ 0.08, and CFI > 0.9 (Schermelleh-Engel, Moosbrugger, & Müller, 2003). Additionally, further hypotheses were tested to evaluate the construct validity of the CQOLC. Those correlations of 0.1 are considered low, those of 0.3 moderate, and those of > 0.5 high (Cohen, Manion, & Morrison, 2005).

#### Hypotheses testing

For the evaluation of construct validity by hypothesis testing and responsiveness, the following four hypotheses were stated on the basis of existing literature before the data analysis.

- H1: The CQOLC correlates positively with global HRQoL. Both instruments are measuring HRQoL; thus, a moderate positive correlation is expected between the CQOLC total score and the two general items of the WHOQOL-BREF.
- H2: The correlation between the CQOLC total score and the WHOQOL-BREF domain for psychological health is higher than with the WHOQOL-BREF domain for physical health. This difference is expected, since the correlation with mental health in comparison to physical health was also higher in the unpublished manual of M. Weitzner.
- H3: The CQOLC has sufficient sensitivity to reveal mean differences between caregivers of patients who receive curative and palliative treatment, respectively. Weitzner and McMillan (1999) confirmed this in the revalidation of the CQOLC.
- H4: According to Padmaja, Vanlalhruaii, Rana, Tiamongla, and Kopparty (2016), it is expected that correlations between change scores of the caregivers’ HRQoL and the patients’ HRQoL correlate at least moderately positively in between the three time points. We understand change scores as the difference of the CQOLC scores between two of the three points in time in each case.

To test these hypotheses, Pearson’s correlation coefficients between the CQOLC total score and the domains of the WHOQOL-BREF were calculated for the assessment of convergent validity. An independent sample *t* test was executed for comparing the means of caregivers of curatively treated patients and palliatively treated patients, respectively. The level of significance was set at 0.05.

#### Responsiveness

Responsiveness is defined as “the ability of an instrument to detect important change over time in the construct to be measured” (Mokkink, Terwee, Patrick, et al., 2010b). Therefore, correlations of the change scores between the CQOLC total score with the parallel results of the patients’ global HRQoL measured by EORTC-QLQ-C30 were calculated for three time points to rate the responsiveness.

## Results

In the validation study of the questionnaire, a total of 212 caregivers (71.4%) who completed the questionnaires at the baseline (CQOLC, WHOQOL-BREF, socio-demographic questionnaire) were included. In Table 1, the sociodemographic and medical characteristics of the sample are described. An inclusion criterion of the CONGO study was that all cancer patients suffer from breast or other gynaecologic neoplasms and receive chemotherapy. 88.7% of the patients (*n =* 188) received curative treatment and 11.3% (*n =* 24) palliative treatment, respectively. 8.5% of the patients were troubled with relapse.

**Table 1:** Sociodemographic and medical characteristics (CONGO study participants)

	*N*	%
Gender		
Male	155	73.1
Female	55	25.9
Age (years)		
*M* (*SD*)	51.7 (13.6)	
Min-max	17–82	
Relationship with cancer patient		
Married	149	70.3
Child	34	16.0
Other family member	10	4.7
Other	12	5.7
living in the same household		
Yes	159	75.0
No	49	23.1
Age of patient (years)		
*M* (*SD*)	53.4 (11.7)	
Min-max	27–81	
Diagnosis of patient		
Breast	185	87.3
Ovary	18	8.5
Uterus	6	2.8
Cervix	2	0.9
Other	1	0.5

### Item descriptives

In a first step of the validation study, we analysed the missing values for each of the items of the German version of the CQOLC.

The following items had over 5% missing values in the sample: item 21 (patient’s eating habits; 5.7%), item 27 (focus on caregiving; 6.6%), item 31 (deterioration of patient; 17%), and item 35 (family interest in caregiving; 15.6%). Table 2 shows the item descriptiveness of the items with substituted means, as used for CFA. The descriptive results of the CQOLC total score are also shown in this table.

**Table 2:** Item descriptives

*Items*	*M*	*SD*	*S*	*SE*	*K*	*SE*
01. Alteration in daily routine	1.21	0.887	0.689	0.167	0.461	0.333
02. Disruption of sleep	1.40	1.071	0.523	0.167	− 0.355	0.333
03. Impact on daily schedule	0.35	0.636	2.193	0.167	5.502	0.333
05. Maintenance of outside activities	1.22	0.958	0.550	0.167	− 0.147	0.333
06. Financial strain	0.50	0.762	1.671	0.167	2.846	0.333
07. Concern about insurance	0.43	0.658	1.777	0.167	4.347	0.333
08. Economic future	0.55	0.808	1.945	0.167	4.584	0.333
09. Death of patient	1.52	1.243	0.655	0.167	− 0.493	0.333
10. Outlook on life	3.25	0.970	− 1.148	0.167	0.229	0.333
11. Level of stress	1.87	1.026	0.249	0.167	− 0.388	0.333
12. Spirituality	3.27	0.883	− 1.266	0.167	1.598	0.333
13. Day-to-day focus	0.84	0.904	1.027	0.167	0.732	0.333
14. Sadness	1.60	0.993	0.482	0.167	− 0.194	0.333
15. Mental strain	1.37	1.018	0.294	0.167	− 0.675	0.333
16. Social support	1.51	1.057	0.308	0.167	− 0.648	0.333
17. Guilt	0.41	0.718	1.912	0.167	3.824	0.333
18. Frustration	0.91	0.982	1.157	0.167	1.066	0.333
19. Nervousness	1.14	1.034	0.693	0.167	− 0.119	0.333
20. Impact of illness on family	1.67	1.152	0.417	0.167	− 0.507	0.333
21. Patient’s eating habits	0.90	1.137	1.345	0.167	1.083	0.333
22. Relationship with patient	1.77	1.220	0.231	0.167	− 0.788	0.333
23. Informed about illness	1.43	0.937	0.221	0.167	− 0.049	0.333
24. Transportation	0.35	0.692	2.254	0.167	4.896	0.333
25. Adverse effects of treatment	1.88	1.114	0.012	0.167	− 0.715	0.333
26. Responsibility for patient’s care	0.66	0.774	0.986	0.167	0.353	0.333
27. Focus of caregiving	1.60	1.105	0.296	0.167	− 0.574	0.333
28. Family communication	1.98	1.032	− 0.133	0.167	− 0.497	0.333
29. Change in priorities	0.77	0.859	0.926	0.167	0.096	0.333
30. Protection of patien	0.72	0.913	1.187	0.167	0.880	0.333
31. Deterioration of patient	1.80	1.135	0.146	0.167	− 0.551	0.333
32. Management of patient’s pain	0.65	0.800	1.269	0.167	1.452	0.333
33. Future outlook	0.97	0.997	1.188	0.167	1.382	0.333
34. Family support	1.33	1.064	0.442	0.167	− 0.290	0.333
35. Family interest in caregiving	0.83	1.023	1.652	0.167	2.526	0.333

*M* mean*, SD* stanard deviation, *S* skewness, *SE* standard error, *K* kurtosis

The CQOLC total scores ranged between 45 and 125. The mean score in the sample was 93.4 with a standard deviation of 14.29. The correlation of items with their intended domain using corrected item-total correlations was considered. All in all, three items reached a poor value < 0.30. Two of these items belong to the subscale positive adaptation. The corrected item-total correlations of item 10 (outlook on life) were 0.29 and of item 12 (spirituality) was 0.12.

### Reliability

The values for internal consistency of the four subscales ranged between 0.60 and 0.83. Table 3 shows the values for internal consistency of the subscales in the sample of 212 caregivers, which are in accordance with the reliability values reported by Mokkink, Terwee, Patrick, et al. (2010b).

**Table 3:** Internal consistency (*N =* 212)

Subscale	Cronbach’s alpha	Number of items
*Burden*	0.83	10
*Disruptiveness*	0.75	7
*Positive adaptation*	0.60	7
*Financial concerns*	0.81	3

### Construct validity

**Fig. 1:**
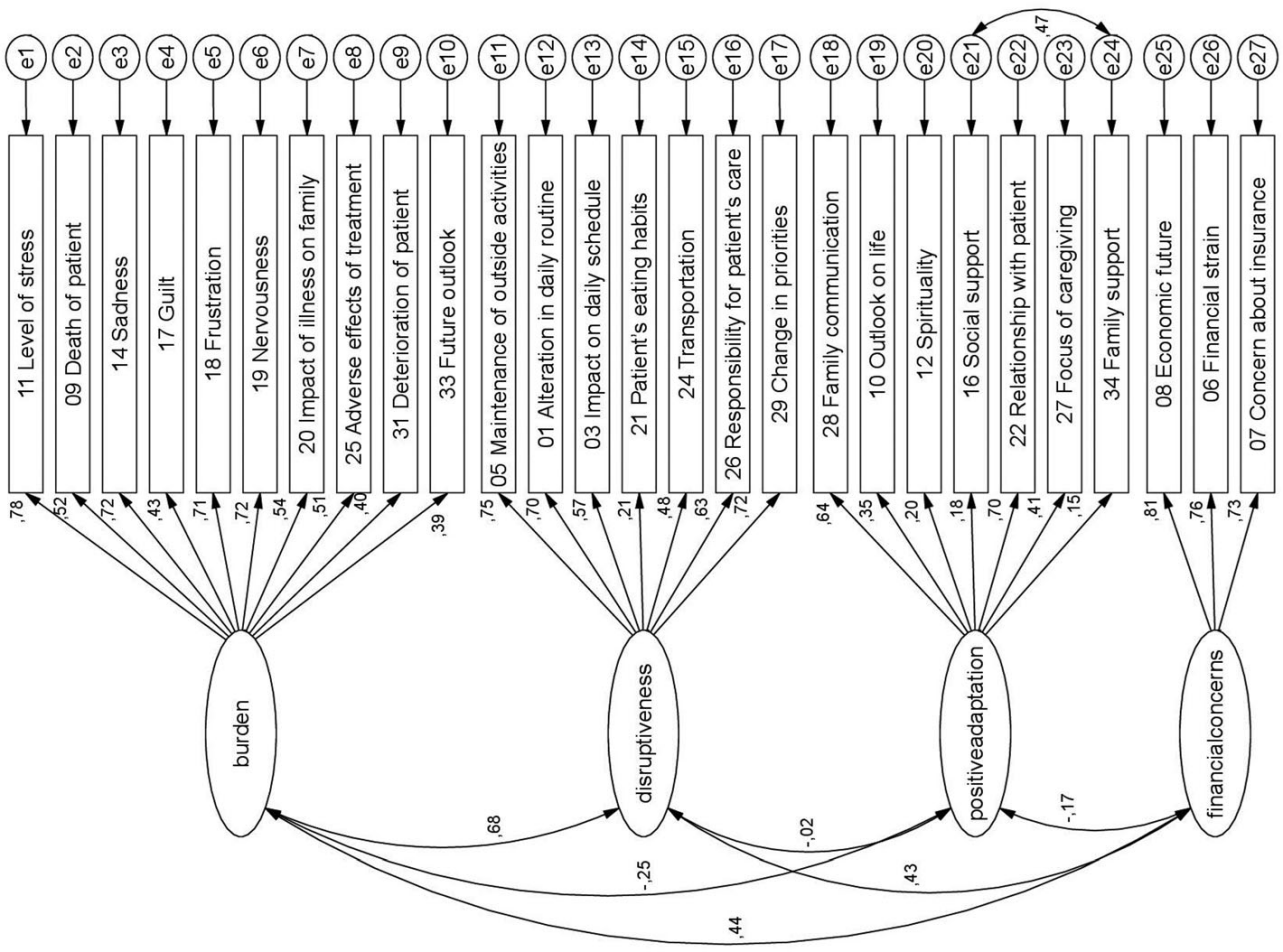
Path model for the CFA of the German CQOLC with residual path coefficients

We conducted a CFA based on the original factor structure provided unacceptable model fit at first: CMIN/DF = 2.12, RMSEA = 0.07 (90% confidence interval 0.06–0.08), and CFI = 0.79. Based on the output of modification indices, a correlation between two items was added to the model to reduce chi square value: item 16 (“I get support from my friends and neighbours.”) and item 34 (“I am satisfied with the support I get from my family.”), both very low in factor loading and both deal with the support of significant others. All factor loadings on their corresponding items were statistically significant. Therefore, and for the confirmatory purpose, no items were removed in the first analysis. Figure 1 shows the path model with the specific modification for the German version of the scale with the corresponding path coefficients. The fit indices for this model resulted in CMIN/D = 1.98, RMSEA = 0.07 (90% confidence interval 0.06–0.08), and CFI = 0.82.

**Fig. 2:**
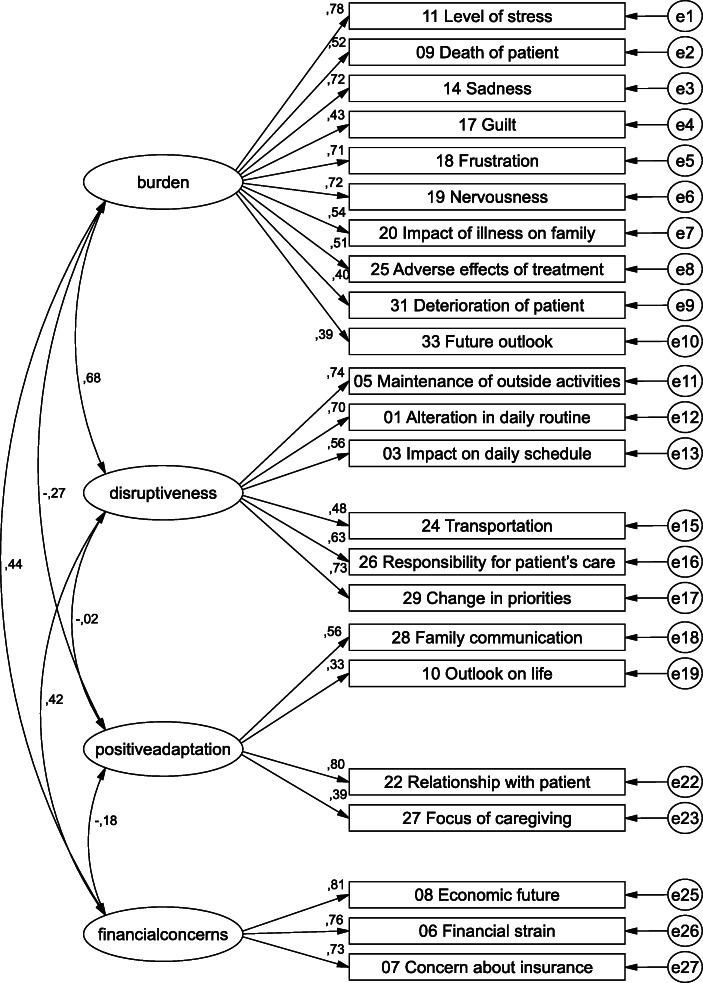
CFA of the German CQOLC without items 21, 12, 16, and 27

We performed another CFA without the items with factor loadings below 0.3 (items 21, 12, 16, 27). The model fit of this model yielded in slightly better but still inacceptable values: CMIN/DF = 1.91, RMSEA = 0.07 (90% confidence interval 0.06–0.08), and CFI = 0.87. Due to the deleted items, the additionally added correlation between the error terms is omitted in this model. See Fig. 2 for the CFA of the German CQOLC without items 21, 12, 16, and 27.

### Hypothesis testing

Table 4 shows the correlations of the CQOLC total score with the WHOQOL-BREF domains. The first two hypotheses used for the validation analyses could be confirmed in this sample.

**Table 4:** Correlations with the CQOLC total score (hypotheses 1 and 2)

	*Global Health* WHOQOL-BREF		*Psycho-logical* WHOQOL-BREF	*Physical Health* WHOQOL-BREF
CQOLC total score	Pearson Correlation	0.47	0.62	0.45
	Sig. (2-tailed)	< 0.001	< 0.001	< 0.001
	95% CI	0.36–0.57	0.53–0.70	0.34–0.55
	*n*	210	209	209

The assumption of homogeneity of variances of the scores in the groups of palliative and curative patients was not given. Instead of a *t* test, the Welch test was performed. The Welch test showed no group difference (*p* = 0.959). Based upon these results, hypothesis 3 could not be confirmed.

### Responsiveness

Pearson’s correlation coefficients for the association between the change scores of the CQOLC total score and patients’ global HRQoL at the three time points were low. They reached *r* = 0.03 between T3 and T1 (*n =* 136), *r* = 0.06 between T4 and T1 (*n =* 135), and *r* = 0.33 between T4 and T3 (*n =* 135). These results did not confirm hypothesis 4.

## Discussion

The main objective of this study was to evaluate the psychometric properties of the German version of the CQOLC, an instrument measuring disease-specific quality of life of cancer patients’ family members, caregivers, or close friends. We obtained several key and novel results. The German version with 34 items has satisfactory internal consistency for three factors ranging between 0.75 and 0.83 and 0.60 for the factor positive adaptation. The assessment of reliability by means of two test halves with Spearman-Brown correction resulted in an acceptable value.

The CFA did not confirm the multi-dimensional structure of the assessment instrument. Four items (item 21, 12, 16, 27) showed factor loadings below 0.3; even without these items, we could not achieve an acceptable model fit. This indicates that the assignment to the factors did not correspond to that of the English version, but that a different factor structure may be more appropriate for the German version. This discrepancy could be attributed to limited objectivity with missing equivalence in a few items. We therefore conclude that the German version of the CQOLC needs to be revised. First of all, the translation and adaptation of the items should be carried out involving cancer caregivers. In a new data collection, the factor structure of the German instrument should be determined and subsequently verified.

The convergent validity was assessed with the WHOQOL-BREF as a global HRQoL instrument, and our hypothesis 2 could be confirmed. The administration of global HRQoL instruments in specific samples, such as caregivers of cancer patients, can be problematic (Grov & Valeberg, 2012). The moderate correlation with global HRQoL may confirm that the CQOLC measures beyond global HRQoL. This is comprehensible because the construct HRQoL is composed differently and focuses on a holistic everyday health experience, while the CQOLC involves specific aspects impacting on one’s health while being involved in the care of a family member affected with cancer. Most of all, caregiver burden is an influential factor for caregivers’ HRQoL (Bahrami & Farzi, 2014; Chua et al., 2016; Rha, Park, Song, Lee, & Lee, 2015; Turkoglu & Kılıc, 2012; Yun et al., 2005). It is therefore likely that measuring caregivers’ HRQoL with the CQOLC offers a certain added value compared to existing translations of global HRQoL instruments. This assumption is also supported by other findings (Lapid et al., 2016; Mahendran et al., 2017; Waldron, Janke, Bechtel, Ramirez, & Cohen, 2013) indicating that caregivers’ HRQoL levels may benefit from dyadic interventions improving cancer patients’ HRQoL, and positive long-term effects can result in specific HRQoL domains (Lapid et al., 2016; Mahendran et al., 2017). This effect on caregivers’ HRQoL could not have been shown in global HRQoL (Lapid et al., 2016). The correlation with psychological health is higher than with physical health, which is congruent with the original publication (Weitzner et al., 1997) and other studies assessing the association between mental health (Chua et al., 2016; Gorji et al., 2012; Wadhwa et al., 2013) and caregiver HRQoL. Rhee et al. (2005) also suggested that the Korean version of the CQOLC reflects the mental health issues better than physical ones. Other statements about cross-cultural validation are hardly possible due to the heterogeneity in the particular CQOLC versions. Known group validity assessment did not provide the result that the CQOLC German version can differ between caregivers of curatively or palliatively treated patients in this sample. This differentiation could possibly not be shown in this study due to the small number of palliatively treated patients in the sample, but this is consistent with the results of a Norwegian study (Grov & Valeberg, 2012) and in a study involving husbands of breast cancer patients (Wagner, Bigatti, & Storniolo, 2006).

Our last hypothesis 4 referred to the responsiveness of the instrument. Shahi et al. (2014) ascertained that better HRQoL of patients is aligned to better HRQoL of caregivers, and Padmaja et al. (2016) found a high and significant correlation between the patients’ and their family caregivers’ HRQoL assessed with the same instruments. Weitzner and McMillan (1999), Shilling, Matthews, Jenkins, and Fallowfield (2016), and Michels, Boulton, Adams, Wee, and Peters (2016) demand the examination of responsiveness in future research. No comparable data are available to determine the degree of the responsiveness of the CQOLC because the responsiveness or sensitivity to change over time of the CQOLC in terms of perceived patients’ HRQoL has not yet been examined. The responsiveness of the CQOLC by means of calculating correlations between change scores with patients’ global HRQoL could not be demonstrated in this sample: The correlation coefficients were in the expected direction but close to zero. A moderate correlation could be stated only after the intervention to the 6-month follow-up. The sample included caregivers of patients in the intervention group as well as the control group. Possible explanations for these results could be the influences beyond the patients’ global HRQoL, response shift, and overall small changes in caregivers’ HRQoL over time. Therefore, it seems that the CQOLC scores in this sample are not responsive to changes in the global HRQoL of the attendant patients. Possible changes in the role of the caregiver caused by a possibly strengthening of the cancer patients through the interventions of the study cannot be detected by the CQOLC (Michels et al., 2016). This could possibly be a further reason for the low correlations between the change scores in this sample. A particular aspect of the study is that the majority of caregivers are male (71.1%), as the patients are all women. Future research regarding responsiveness as a measurement property in general should be conducted. A re-evaluation of the wording and the assessment of the relevance of each of the items by caregivers themselves could, however, result in acceptable results for construct validity assessed by CFA.

The original instrument was developed with caregivers of patients diagnosed with various kinds of cancer in different settings. Therefore, the items consist of relevant aspects from their point of view (Weitzner et al., 1997). Since there was no pre-test of the German version, the relevance and the comprehensiveness of the items were only assessed by the project team (*n =* 6). Further reasons for a revision of the items are that a significant amount of time has passed since the conception of the original instrument and that healthcare systems in the country of origin and Germany are different. After this appraisal, it is quite likely that small refinements should be made for better understanding. These refinements could result in even better values for model fit, higher path coefficients, less missing values, and a more adequate assessment of cancer caregiver’s HRQoL.

We were able to report on the most relevant aspects of the COSMIN checklist (Mokkink, Terwee, Knol, et al., 2010; Mokkink, Terwee, Patrick, et al., 2010a, 2010b), although other methods like total omega instead of alpha would be more informative for reliability assessment. We were able to assess some important psychometric properties of a disease-specific HRQoL measurement, namely, the CQOLC, in cancer caregivers in a German context. Another strength was the access to cancer patients’ caregivers, and that not only family members but also other significant persons of cancer patients are involved in this sample. These other closely involved persons may become even more relevant in future patient care if family structures change.

## Conclusions

Based on this validation study with the German translation of the CQOLC, construct validity in the confirmatory factor analysis and internal consistency could not be confirmed. Neither, we could confirm the hypotheses for responsiveness and known groups validity. These results are probably caused by the inequality of the translation and the missing assessment of content validity by caregivers. We recommend to revise the German version of the CQOLC with cancer caregivers and to perform further analyses in the future.

As there are interdependent relationships between the HRQOL detriments and supportive needs between cancer patients and their caregivers, there is a need for a sound disease-specific measurement instrument of caregivers’ HRQoL highlighted in the context of this validation study.

## Supplementary information

**Additional file 1.** Item wording of the original CQOLC and the German version.

## Data Availability

The datasets used and analysed during the current study are available from the corresponding author on reasonable request.

## Abbreviations

CFA: Confirmatory factor analysis
CFI: Bentler Comparative Fit Index
CMIN/df: Chi square value/degrees of freedom
CONGO: Complementary Nursing in Gynaecologic Oncology
COSMIN: Consensus-based Standards for the selection of health status Measurement Instruments
CQOLC: Caregiver Quality of Life Index-Cancer
EORTC-QLQ-C30: European Organization for Research and Treatment of Cancer Quality of Life Questionnaire
HRQoL: Health-related quality of life
MOS SF-36: Medical Outcomes Study Short Form 36
PROQOLID: Patient-Reported Outcome and Quality of Life Instruments Da-tabase
QOL: Quality of life
RMSEA: Root mean square error of approximation
WHOQOL-BREF: World Health Organization Quality of Life short version
