# Supplementary material for: Validation of a German version of the Caregiver Quality of Life Index-Cancer (CQOLC) in a sample of significant others of breast and gynaecologic cancer patients
Source: Psicol Reflex Crit. 2020 Jul 20;33:15. doi: 10.1186/s41155-020-00155-8 (PMC7371772; doi:10.1186/s41155-020-00155-8)
Supplement: Supplementary file 1 — Additional file 1. Item wording of the original CQOLC and the German version. [file 41155_2020_155_MOESM1_ESM.docx]

**Additional file 1** – Item wording of the original CQOLC and the German version

|  | Original English version of the CQOLC | Item in the German version used for the validation study |
| --- | --- | --- |
| 1 | It bothers me that my daily routine is altered. | Es belastet mich, dass mein üblicher Tagesablauf verändert ist. |
| 2 | My sleep is less restful. | Mein Schlaf ist weniger erholsam. |
| 3 | My daily life is imposed upon. | Ich fühle mich in meinem täglichen Leben ausgenutzt. |
| 4 | I am satisfied with my sex life. |  |
| 5 | It is a challenge to maintain my outside interests | Es ist schwierig, anderen Interessen weiterhin gerecht zu werden. |
| 6 | I am under a financial strain. | Ich stehe unter finanziellem Druck. |
| 7 | I am concerned about our insurance coverage. | Ich mache mir Sorgen um unseren Versicherungsschutz. |
| 8 | My economic future is uncertain | Meine wirtschaftliche Zukunft ist unsicher. |
| 9 | I fear my loved one will die. | Ich fürchte, dass der von mir geliebte Mensch sterben wird. |
| 10 | I have more of a positive outlook on life since my loved one's illness. | Ich habe eine positivere Einstellung, seitdem er/sie erkrankt ist. |
| 11 | My level of stress and worries has increased. | Stress und Sorgen haben sich für mich spürbar verstärkt. |
| 12 | My sense of spirituality has increased. | Spiritualität hat einen größeren Raum in meinem Leben eingenommen. |
| 13 | It bothers me, limiting my focus to day-to-day. | Es belastet mich, mich darauf beschränken zu müssen, mich auf einen Tag nach dem anderen zu konzentrieren. |
| 14 | I feel sad. | Ich bin traurig. |
| 15 | I feel under increased mental strain. | Ich fühle mich zunehmend mental unter Stress. |
| 16 | I get support from my friends and neighbours. | Ich erfahre die Unterstützung meiner Freunde und Nachbarn. |
| 17 | I feel guilty. | Ich habe Schuldgefühle. |
| 18 | I feel frustrated. | Ich empfinde Frustration. |
| 19 | I feel nervous. | Ich bin nervös. |
| 20 | I worry about the impact my loved one's illness has had on my children or other family members. | Ich mache mir Sorgen über mögliche Auswirkungen der Krankheit dieses von mir geliebten Menschen auf meine Kinder bzw. auf andere Familienangehörige. |
| 21 | I have difficulty dealing with my loved one's changing eating habits. | Mit seinen/ihren veränderten Essgewohnheiten kann ich nicht gut umgehen. |
| 22 | I have developed a closer relationship with my loved one. | Es ist ein engeres Verhältnis zu ihm/ihr entstanden. |
| 23 | I feel adequately informed about my loved one's illness. | Ich fühle mich über die Krankheit ausreichend informiert. |
| 24 | It bothers me that I need to be available to chauffeur my loved one to appointments. | Es belastet mich, dass ich als Chauffeur zur Verfügung stehen muss. |
| 25 | I fear the adverse effects of treatment on my loved one. | Ich fürchte die negativen Auswirkungen der Behandlung bei ihm/ihr. |
| 26 | The responsibility I have for my loved one's care at home is overwhelming. | Die Verantwortung, die ich für seine/ihre Pflege zu Hause trage, übersteigt meine Kräfte. |
| 27 | I am glad that my focus is on getting my loved one well. | Ich bin froh, dass ich mich ganz auf sein/ihr Wohlbefinden konzentriere. |
| 28 | Family communication has increased. | Die Kommunikation in der Familie hat zugenommen. |
| 29 | It bothers me that my priorities have changed. | Es belastet mich, dass meine Prioritäten sich verändert haben. |
| 30 | The need to protect my loved one bothers me. | Die Notwendigkeit, diesen Menschen zu schützen, belastet mich. |
| 31 | It upsets me to see my loved one deteriorate. | Es geht mir nah, zu sehen wie sie/er immer weniger wird. |
| 32 | The need to manage my loved one's pain is overwhelming. | Die Notwendigkeit mich um seine/ihre Schmerztherapie zu kümmern, belastet mich. |
| 33 | I am discouraged about the future. | Ich bin pessimistisch, was die Zukunft betrifft. |
| 34 | I am satisfied with the support I get from my family. | Ich bin mit der Unterstützung, die ich von meiner Familie erfahre, zufrieden. |
| 35 | It bothers me that other family members have not shown interest in taking care of my loved one. | Es stört mich, dass andere Familienangehörige kein Interesse an der Pflege dieses mir nahestehenden Menschen gezeigt haben. |
